# Perforin, granzyme B, and FasL expression by peripheral blood T lymphocytes in emphysema

**DOI:** 10.1186/1465-9921-8-62

**Published:** 2007-09-06

**Authors:** Mathieu C Morissette, Julie Parent, Julie Milot

**Affiliations:** 1 Hôpital Laval, Institut de cardiologie et de pneumologie, Québec, Qc, Canada

## Abstract

**Background:**

It is generally accepted that emphysematous lungs are characterized by an increase in the numbers of neutrophils, macrophages, and CD8^+ ^T lymphocytes, the lasts having increased cytotoxic activity. Because systemic inflammation is also a component of emphysema, we hypothesize that peripheral CD8^+ ^T lymphocytes of emphysematous smokers who show evidence of systemic inflammation will have higher expression of cytotoxic molecules.

**Methods:**

We assessed parameters of systemic inflammation in normal individuals (smokers or non-smokers) and in emphysematous subjects with an active smoking history by measuring serum interleukine-6, C-reactive protein, and tumor necrosis factor. Expression of perforin, granzyme B, and FasL protein by CD8^+ ^T lymphocytes, CD4^+ ^T lymphocytes, and natural killer cells were assessed by flow cytometry while perforin, granzyme B, and FasL mRNA expression were measured on purified systemic CD8^+ ^T lymphocytes by real-time PCR.

**Results:**

Emphysematous smokers had higher levels of serum interleukine-6 than normal subjects. Even with the presence of systemic inflammation in emphysematous smokers, the percentage of peripheral CD8^+ ^T lymphocytes, CD4^+ ^T lymphocytes, and NK cells expressing perforin and granzyme B protein was not different between the three groups.

**Conclusion:**

Despite evidence of systemic inflammation, peripheral T lymphocytes of emphysematous smokers did not show higher levels of cytotoxic markers, suggesting that increase of activated T lymphocytes in the emphysematous lung may be due to either activation in the lung or specific peripheral recruitment.

## Background

Emphysema is a major component of chronic obstructive pulmonary disease (COPD) which is essentially induced by cigarette smoking or exposure to other noxious gases. It is characterized by an inflammation-mediated destruction of the lung parenchyma which leads to distal air space enlargement and non-fully reversible airflow obstruction [1]. Neutrophils, macrophages, and T lymphocytes are thought to play an important role in this inflammatory process [2].

Because the presence of T lymphocytes in smoker's lungs has been found to correlate with emphysema [3], their role in the pathogenesis of the disease has been studied extensively. In emphysematous subjects, the number of CD8^+ ^T lymphocytes is increased in central [4] and peripheral airways [5-7], leading to a decrease of the CD4/CD8 ratio [4,5,7]. Majo *et al*. [5] also showed that there was a correlation between the presence of CD8^+ ^T lymphocytes in the lung parenchyma of smokers and their smoking history as well as the apoptotic index of their alveolar cells. Chrysofakis *et al*. [8] were able to document that perforin expression, a cytotoxic and activation marker, and cytotoxic activity of CD8^+ ^T lymphocytes in the sputum of COPD smokers were higher than in smokers without COPD. All these data suggest that CD8^+ ^T lymphocytes may have an important role in the pathogenesis of emphysema. However, emphysema's damages are not confined to the lung. Indeed, important systemic effects such as muscle wasting and cachexia are also part of the disease [9,10].

Although several authors have demonstrated the importance of lung T lymphocytes in emphysema, few studies have described their state of activation and their cytotoxic potential in the peripheral blood. Perforin, granzyme B, and FasL are cytotoxic molecules used by CD8^+ ^T lymphocytes and natural killer (NK) cells to induce apoptosis [11]. Constitutively expressed by NK cells, perforin, granzyme B, and FasL expression is induced upon activation by antigen presenting cells, such as dendritic cells, in CD8+ T lymphocytes [11]. Because increased expression of perforin and granzyme B by peripheral blood CD8^+ ^T lymphocytes has been reported in other chronic diseases such as asthma [12] and systemic lupus erythematosus [13], overexpression of cytotoxic molecules by systemic CD8^+ ^T lymphocytes or other types of lymphocytes may also lead to non-specific systemic damages in the lungs of emphysematous subjects. In the current study, we postulated that systemic CD8^+ ^T lymphocytes of emphysematous smokers with evidence of systemic inflammation will show higher expression of cytotoxic and activation markers such as perforin, granzyme B, and FasL than that of subjects with normal lung function. To test this hypothesis, we measured serum interleukine-6 (IL-6), tumor-necrosis factor (TNF), and C-reactive protein (CRP) in smokers with emphysema, smokers without emphysema, and in non smokers with normal lung function. Expression of perforin, granzyme B, and FasL protein by systemic T lymphocytes and NK cells was compared between the three groups.

The results show that despite evidence of low-grade systemic inflammation in emphysematous smokers, the peripheral T lymphocytes of those subjects do not express higher levels of perforin, granzyme B, and FasL, suggesting that the increase of activated T lymphocytes in the lung of emphysematous subjects may be due to either local intra pulmonary activation or to specific recruitment.

## Methods

### Subjects

Twenty-nine subjects matched for age and sex were included in the study: nine were active smokers with evidence of emphysema and airflow limitation defined according to the Global Initiative for Chronic Obstructive Lung Disease (GOLD) [1] (FEV_1 _< 80% predicted value, FEV_1_/FVC < 70% predicted value, and reversibility ≤ 12% and 200 ml after salbutamol inhalation), 10 were smokers with normal lung function, and 10 were non-smokers with normal lung function. In subjects with airflow limitation, the presence of emphysema was further confirmed by a radiologist following CT scanning analyses and a diffusion capacity (DLCO) < 80% predicted value for man and < 70% predicted value for women. Subjects were excluded from the study if they have a history of cancer in the past five years, active liver, heart or kidney disease, diabetes or any other non emphysematous pulmonary condition. In addition, all emphysematous subjects had to have a negative prick test for common allergens and they had been free of disease exacerbation within the previous 3 months. Two subjects were taking inhaled steroids but none had received oral steroids or antibiotics for at least four weeks before the study. The study was approved by the Hospital Research Ethic Committee and all subjects gave a written consent.

### Serum IL-6, TNF, and CRP quantification

Serum TNF and IL-6 levels were measured with the Quantikine HS human TNF immunoassay and the Quantikine HS human IL-6 immunoassay (R&D Systems Inc.; Minneapolis, MN) according to the manufacturer's instructions. Serum CRP levels were measured by a high sensitivity nephelometer immunoassay using Dabe Behring BN Prospec instrumentation (Dabe Behring, Deerfield, IL, USA).

### Peripheral blood mononuclear cells isolation and CD8^+ ^T lymphocytes purification

**Figure 1:**
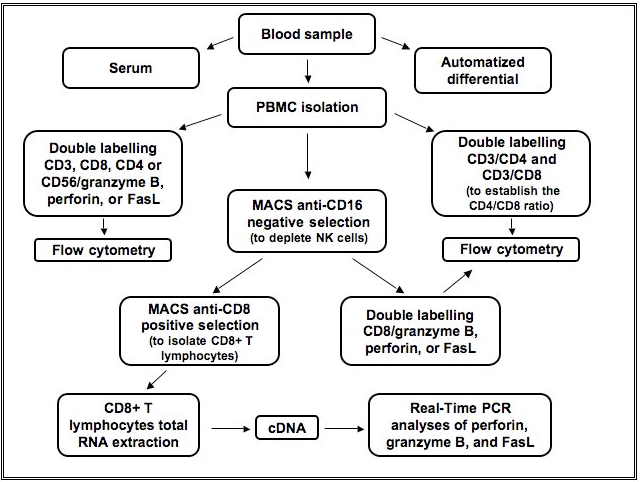
Blood processing protocol.

For each subject, approximately 150 ml of blood was drawn on K_3_-EDTA in BD Vacutainer (BD Biosciences; Mississauga, ON, Canada) and processed as shown in Figure 1. Five milliliters of blood was used for automatic differential analysis of blood cells, another 20 milliliters was centrifuged at 2000 rpm for 10 min, the supernatant being collected and kept at -80°C for serum IL-6, TNF, and CRP quantification, and the remaining was used for peripheral blood mononuclear cells (PBMC) isolation using Ficoll-Paque Plus density gradient (Amersham Biosciences; Piscataway, NJ) according to the manufacturer's instructions. A fraction of the PBMC was directly used for flow cytometry analysis. To deplete NK cells (CD16^+^) that are faintly CD8^+^, the remaining PBMC were labeled with mouse anti-human CD16 conjugated to magnetic beads (Miltenyi Biotec Inc.; Auburn, CA) and CD16^- ^cells were negatively selected. Some of these CD16^- ^cells were then used for flow cytometry analysis while the remaining cells were labeled with mouse anti-human CD8 conjugated to magnetic beads (Miltenyi Biotec Inc.). CD8^+ ^T lymphocytes were positively selected with a purity greater than 90% as documented by flow cytometry (CD3^+^/CD8^+ ^cells) (data not shown). Total RNA was extracted from CD8^+^/CD16^- ^T lymphocytes. Analyses were done to ensure that the magnetic cells sorting (MACS) and binding of CD8 antigen did not activate the CD8^+ ^T lymphocytes: indeed, the percentage of perforin^+^/CD8^+ ^and granzyme B^+^/CD8^+ ^cells was determined before CD8^+ ^cells selection. Following the positive selection, the resulting CD8^+ ^cells were cultured at 37°C 5%CO_2 _in RPMI 1640 without serum for up to 24 hours. After that period of time, the percentage of perforin^+^/CD8^+ ^and granzyme B^+^/CD8^+ ^cells was determined. No modulation in perforin nor granzyme B protein expression was observed either before and after the CD8 selection (data not shown).

### Double labeling of T lymphocyte subsets and flow cytometry analyses

The following mouse anti-human monoclonal antibodies were used to label PBMC and/or CD16^- ^cells: phycoerythrin-cyanine (PE-Cy5)-conjugated anti-CD3 (clone HIT3a)(T lymphocytes), fluorescein isothiocyanate (FITC)-conjugated anti-CD3 (clone HIT3a)(T lymphocytes), PE-Cy5-conjugated anti-CD8 (clone HIT8a)(CD8^+ ^T lymphocytes), PE-Cy5-conjugated anti-CD4 (clone RPA-T4)(CD4^+ ^T lymphocytes), phycoerythrin (PE)-conjugated anti-CD56 (clone B159)(Natural Killer cells), FITC-conjugated anti-perforin (clone δG9), FITC-conjugated anti-granzyme B (clone GB11), and Alexafluor 488-conjugated anti-FasL (clone NOK-1) (BD Biosciences). The appropriate conjugated mouse isotype controls were used in parallel (BD Biosciences).

To label surface antigens (CD3, CD8, CD4, CD56, and FasL), 0.5 × 10^6 ^cells in phosphate buffered saline solution (PBS) supplemented with 1% bovine serum albumine (BSA) (Sigma-Aldrich Canada Ltd; Oakville, ON, Canada) were incubated with appropriate antibodies or isotype controls for 40 min on ice and protected from light. For intracellular labeling of perforin or granzyme B, cells were further permeabilized using Cytofix/Cytoperm solution (BD Biosciences) for 20 min on ice and protected from light. Cells were then washed with PBS-BSA 1% containing 0.1% of saponin (Sigma-Aldrich Canada Ltd) and then incubated with the appropriate antibody or isotype control 40 min on ice and protected from light. Cells were finally washed with PBS-BSA 1%-saponin 0.1% and immediately analyzed by flow cytometry.

The labeled cells were analyzed on a Coulter EPICS XL-MCL flow cytometer (Beckman-Coulter; Mississauga, ON, Canada) with the EXPO 32 APC XL 4 Color program (Beckman-Coulter). The lymphocytes were tightly gated using complexity (side-light scatter) and volume (forward-light scatter) parameters. The percentages of perforin, granzyme B, or FasL positive cells for each population (CD3^+^, CD8^+^, CD4^+ ^or CD56^+^) and the CD4/CD8 ratio were evaluated by two colors analysis. At least 10^5 ^cells were analyzed in each session.

### Real-time PCR

After magnetic cell sorting of peripheral blood CD8^+ ^T lymphocytes (CD16^-^/CD8^+^), total RNA was extracted using RNeasy mini kit (Qiagen Inc.; Mississauga, ON, Canada) according to the manufacturer's instructions. To eliminate DNA contamination, extracted RNA was then treated with RQ1 DNase (Promega Inc.; Ottawa, ON, Canada) for 30 min at 37°C. Resulting RNA was washed and concentrated in 37 μl of water using Microcon 100 filtration column (Millipore; Billerca, MA, USA) and quantified by spectrophotometry using GeneQuant pro RNA/DNA Calculator (Biochrom Ltd; Cambridge, United Kingdom). Total RNA (500 ng) was reversed transcribed with Omniscript Reverse Transcriptase (Qiagen Inc.) according to the manufacturer's specification and using random octamers. Resulting cDNA (final volume of 20 μl) was kept at -80°C until further utilization.

Real-time PCR analyses were performed on a DNA Engine Opticon 2 System (MJ Research; Reno, NV) with the QuantiTect SYBR Green PCR Kit (Qiagen Inc.) according to the manufacturer's specification. Primers sequences are shown in Table 1 and were designed according to Lyon *et al*. [14] (18S rRNA, used as housekeeping gene [15]) and Wang and Seed [16] (perforin, granzyme B, and FasL). PCR efficiency was confirmed for all genes to ensure equivalent amplification rates when compared to 18S rRNA. For each PCR reaction, 1 μl of the 1/100 dilution of the cDNA stock was used for perforin, granzyme B, and FasL analyses, and 1 μl of the 1/1000 dilution was used for 18S rRNA analysis. The PCR conditions were 94°C for 15 min, then 35 cycles at 94°C for 15 sec, 30 sec at specific annealing temperature (see Table 1) and 72°C for 30 sec.

**Table 1:** Real-time PCR primers description

**Gene**	**GenBank accession number**	**Primer sequences**	**Amplicon size**	**Annealing temperature**
**18S rRNA**	NR_003286	F: 5'-TGCATGGCCGTTCTTAGTTG-3'	76 bp	57°C
		R: 5'-AGTTAGCATGCCAGAGTCTCGTT-3'		
**Perforin**	A26435	F: 5'-CGCCTACCTCAGGCTTATCTC-3'	155 bp	58°C
		R: 5'-CCTCGACAGTCAGGCAGTC-3'		
**Granzyme B**	NM_004131	F: 5'-TGGGGGACCCAGAGATTAAAA-3'	100 bp	55°C
		R: 5'-TTTCGTCCATAGGAGACAATGC-3'		
**FasL**	AF288573	F: 5'-TCAATGAAACTGGGCTGTACTTT-3'	101 bp	58°C
		R: 5'-AGAGTTCCTCATGTAGACCTTGT-3'		

The method used to quantify the variation of expression for perforin, granzyme B and FasL mRNA was the 2^ΔΔCt ^method [17]. Based on the 18S rRNA crossing threshold (Ct), the ΔCt of each gene was determined for each subject. Then, for each group of subjects, the mean expression of each gene was compared to the normal non-smoker group who represents the baseline expression.

### Data analyses

Data are expressed as mean ± standard error (SEM). The three groups were compared with the analysis of variance (ANOVA) followed, if *p *< 0.05, by a *post hoc *Fisher's probable least-squares difference (PLSD) test. Smoking history of smokers with and without emphysema was compared with an unpaired Student's T test (significantly different if *p *< 0.05).

## Results

### Clinical findings

The characteristics of subjects are presented in Table 2. The mean age of subjects included in each of the three groups was similar. Smokers with emphysema had severe airflow obstruction with a mean FEV_1 _of 40% predicted (17%–64%) and a mean ratio FEV_1_/FVC of 45% (32%–64%) whereas normal smokers and non-smokers had normal lung function. Diffusion (DLCO) was reduced in smokers with emphysema with a mean of 53% (30%–81%) of predicted value. Smokers with emphysema had a more important smoking history (70 pack-years, range 39–113) than normal smokers (43 pack-years, 16–65) and a lower body mass index (BMI) than normal smokers and non-smokers. According to the GOLD index for COPD [1], two emphysematous smokers were classified as GOLD stage II, five as GOLD stage III, and two as GOLD stage IV.

**Table 2:** Subjects characteristics

**Variables**	**Normal non-smokers n = 10**	**Normal smokers n = 10**	**Emphysematous smokers n = 9**
**Age, yr**	62 ± 2	58 ± 1	60 ± 1
**Gender, F/M**	3/7	2/8	2/7
**FEV_1_, % predicted**	112.2 ± 4.6	111.1 ± 4.1	39.6 ± 5.0*
**FEV_1_/FVC**	95.6 ± 1.8	94.0 ± 5.8	44.6 ± 3.5*
**GOLD stage I/II/III/IV**	-	-	0/2/5/2
**DLCO, % predicted**	-	-	53.4 ± 17.3
**Smoking history, pack-years**	-	43 ± 4	70 ± 8*
**BMI (kg/m^2^)**	26.5 ± 1.1	27.0 ± 1.6	22.2 ± 0.6*
Presence of emphysema**, subjects	-	-	9

* Significantly different from normal smokers and non-smokers (p < 0.05)

** Confirmed by CT scanning

### Serum IL-6, TNF, and CRP

**Figure 2:**
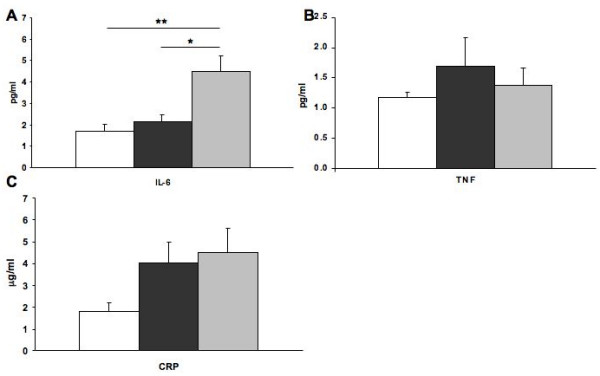
**Serum IL-6, TNF, and C-reactive protein levels**. For each subject, serum levels of A) IL-6 and B) TNF were quantified by ELISA. C) Serum CRP levels were quantified by high sensitivity nephelometry. Columns represent mean ± SEM. *p = 0.002; **p = 0.0003. Normal non-smokers (white), normal smokers (black), and emphysematous smokers (grey).

IL-6 was significantly higher in smokers with emphysema than in the other two groups (Figure 2A) while TNF values were similar in all groups (Figure 2B), and CRP tended to be higher in smokers with or without emphysema (ANOVA, *p *= 0.077) (Figure 2C). Even if this difference between smokers with or without emphysema and non-smokers did not reach statistical level, a mean CRP level above 3 mg/L is considered to be consistent with the presence of low-grade systemic inflammation in emphysematous and normal smokers [18].

### Distribution of peripheral blood inflammatory cells

**Figure 3:**
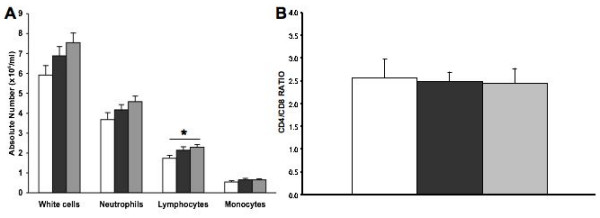
**Blood inflammatory cells counts and CD4/CD8 ratio**. A) Distribution of systemic inflammatory cells in the three groups obtained by automatized cell count. B) PBMC were isolated from each subjects, double labelled with fluorochrome-conjugated antibodies (CD3/CD4 and CD3/CD8), and analysed by flow cytometry. For each subject, CD4/CD8 ratio was obtained by dividing the %CD3^+^/CD4^+ ^cells by the %CD3^+^/CD8^+ ^cells. Columns represent mean ± SEM. *p = 0.02. Normal non-smokers (white), normal smokers (black), and emphysematous smokers (grey).

There were no significant differences in the total white cell count and in the absolute number of monocytes and neutrophils between each group (Figure 3A). All subjects had normal lymphocytes counts although the absolute number of lymphocytes was significantly higher in emphysematous smokers than in normal non-smokers, and it tended to be higher in normal smokers than in normal non-smokers. Flow cytometry analyses of the CD4/CD8 ratios (Figure 3B) did not show any difference between the three groups.

### Perforin, granzyme B, and FasL protein expression in peripheral lymphocyte subsets

**Figure 4:**
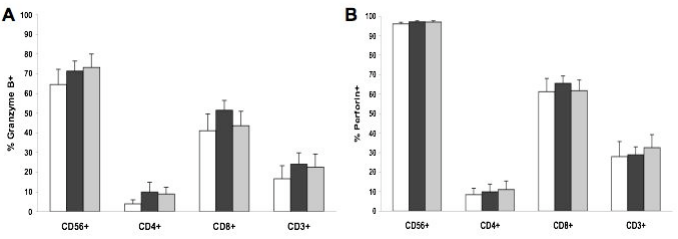
**Perforin and granzyme B protein expression by peripheral CD56+, CD3+, CD4+, and CD8+ cells**. PBMC were isolated from each subjects. They were first labelled with fluorochrome-conjugated anti-CD56, CD4, CD8, or CD3, permeabilized, then labelled with fluorochrome-conjugated anti- A) granzyme B or B) perforin and analysed by flow cytometry. Columns represent mean ± SEM. Normal non-smokers (white), normal smokers (black), and emphysematous smokers (grey).

PBMCs were analyzed by flow cytometry to look at the percentage of CD56^+^, CD4^+^, CD8^+^, and CD3^+ ^cells that express perforin and granzyme B protein (Figure 4), and no difference was observed between the three groups. Further analyses of perforin and granzyme B protein expression on CD8^+ ^cells following CD16^+ ^cells depletion also did not show any differences (data not shown). The mean fluorescence of perforin or granzyme B expressing cells was not significantly different between the groups (data not shown). Protein expression of FasL was not detected by flow cytometry.

### Perforin, granzyme B, and FasL mRNA expression in peripheral CD8^+ ^T lymphocytes

**Figure 5:**
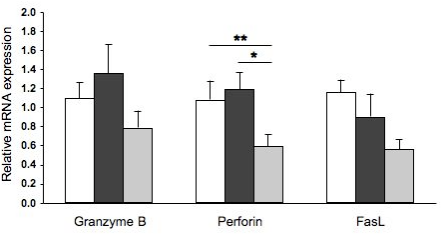
**Perforin, granzyme B, and FasL mRNA expression by isolated peripheral CD8+/CD16- cells**. For each subject, total RNA was extracted from magnetically isolated CD8^+^/CD16^- ^cells. mRNA expression of granzyme B, perforin, and FasL was assessed by real-time PCR, using 18S rRNA as housekeeping gene and compared using 2^-ΔΔCt ^method. Columns represent mean ± SEM. *p = 0.02; **p = 0.05. Normal non-smokers (white), normal smokers (black), and emphysematous smokers (grey).

Expression of perforin, granzyme B, and FasL mRNA was assessed on CD8^+^/CD16^- ^purified cells by real-time PCR (Figure 5). A significant two fold decrease in perforin mRNA was observed in emphysematous smokers when compared to normal smokers and non-smokers. No significant differences were seen in granzyme B and FasL mRNA levels between the groups.

## Discussion

This study was designed to determine if, in the presence of systemic inflammation, the expression of the cytotoxic and activation markers, perforin, granzyme B, and FasL, by peripheral CD8^+ ^T lymphocytes was upregulated in emphysema. We chose to focus on emphysema rather than in other component of COPD such as chronic bronchitis because the alveolar degradation that occurs in emphysema has been linked to CD8+ T lymphocytes activation [5] which is not the case in chronic bronchitis and other types of small airway disease. Despite the presence of low-grade systemic inflammation shown by elevated serum IL-6 and CRP levels (> 3 mg/L) [18] (Figure 2) in emphysematous smokers, the percentage of systemic CD56^+^, CD4^+^, CD8^+^, and CD3^+ ^cells expressing perforin and granzyme B protein was not different between the three groups.

Up to now, fundamental research on emphysema has mainly focussed on the inflammatory reaction that occurs in the lung. The number of CD8^+ ^T lymphocytes have been shown to be increased in the emphysematous lung leading to a decrease in the CD4/CD8 ratio [4,5,7], and possibly to the decline of FEV_1 _[4-7]. In addition, sputum CD8+ cells overexpress perforin and have an increased cytotoxic activity [8]. All of these data suggest that CD8^+ ^T lymphocytes are involved in the inflammation that occurs in the emphysematous lung. Surprisingly, this increase in the number of T lymphocytes in the lung of emphysematous subjects does not seem to be reflected in peripheral blood. Similar to other previously published studies [19,20], we were able to demonstrate that peripheral blood CD4/CD8 ratios (Figure 3B) is not decreased in emphysema, illustrating some degree of discordance between lung and peripheral blood. The expression of several activation markers, such as CD25 [21], CD28 [20], CD29 [20], CD45RO [20], and HLA-DR [19,21], by systemic CD4^+ ^and CD8^+ ^T lymphocytes was also found to be similar between COPD smokers, normal smokers, and normal non-smokers. In an interesting study, Koch *et al*. [22] reported that CD28 expression, which is an important co-stimulatory molecule produced by systemic CD8^+ ^T lymphocytes, was higher in smokers with or without COPD as compared to normal non-smokers subjects. These authors also showed that CD8^+ ^T lymphocytes with a cytotoxic effector phenotype (CD27^-^/CD45RA^+^) were increased in the blood of smokers, with or without COPD when compared to normal non-smokers. These observations suggest that cigarette smoking alone could not only increase the expression of CD28 on systemic CD8^+ ^T lymphocytes but also promote the presence of systemic CD8^+ ^T lymphocytes exhibiting a cytotoxic effector phenotype. In the current study, we analyzed perforin, granzyme B, and FasL because such molecules are known to modulate the cytotoxic power of CD8^+ ^T lymphocytes [11]. Our findings indicate that CD8^+ ^T lymphocytes in the blood of emphysematous smokers do not express more cytotoxic molecules when compared to that of normal smokers and to normal non-smokers. Hodge *et al*. [23] also looked at perforin and granzyme B expression by peripheral blood T lymphocytes, and unlike the findings of this study and of others on the expression of activation markers by peripheral T lymphocytes [19-21], they showed that the percentage of peripheral CD3^+ ^and CD8^+ ^cells expressing perforin and granzyme B is greater in COPD than in normal subjects. Their subjects recruitment was however only based on FEV_1 _and FEV_1_/FVC values and their subjects were not well-characterized in terms of COPD features (emphysema, chronic bronchitis or small airways disease), systemic inflammation, and matched controls. It is possible that with such bias, they recruited subjects with an asthmatic component, which are known to have higher levels of peripheral CD3^+ ^and CD8^+ ^cells expressing perforin [24].

We observed a decrease in the perforin mRNA levels in CD8^+^/CD16^- ^T lymphocytes of emphysematous smokers compared to that of normal smokers (Figure 5), although this difference was not reflected in the percentage of cell expressing perforin (Figure 3B) or in the mean fluorescence. This discordance between mRNA and protein levels may be explained by the fact that a two fold decrease in the perforin mRNA is insufficient to influence the perforin protein level in the cell, that some post-transcriptional modifications are involved, or that perforin is contained and accumulated in granules which may interfere with the quantitative relation between mRNA and protein into the cell.

The results of this study come from a highly selected population of emphysematous subjects who had some evidence of systemic inflammation, as shown by high serum IL-6 and CRP levels (Figure 2A–C). The absence of modulation in the serum TNF levels (Figure 2B), also observed by Debigaré *et al*. [25], could be explained by the fact that TNF is mostly increased during periods of exacerbation of the disease or during the cachexia process [26,27]. In our study, emphysematous subjects did not have exacerbation of their disease for at least three months prior to analysis and all and had normal weight despite a significant lower BMI when compared to the other groups. However, this systemic inflammation in emphysematous smokers (Figure 2) was not associated with an increase in CD8^+ ^T lymphocytes cytotoxic markers.

This difference between blood and lung cell populations in emphysematous subjects was not only observed in the lymphocyte population but also in monocytes/macrophage which are other important cells in the physiopathology of emphysema [2]. These do not appear to be increased or more activated in emphysematous subjects' blood [28] despite the fact that alveolar macrophages are markedly increased and highly activated in the parenchyma of emphysematous lungs, and that they release much more proteases than in normal smokers and non-smokers [29,30].

Our data suggest that activated CD8^+ ^T lymphocytes in the emphysematous lung may not be due to an increase in markers of activation by peripheral blood CD8^+ ^T lymphocytes and that a specific recruitment of inflammatory cells by the lung due to specific chemotactic signals might be a better hypothesis. Indeed, Saetta *et al*. [31] found an increase of CXCR3^+ ^cells, a Th1 receptor for IFN-γ-induced chemokines, and IP-10, a CXCR3 ligand, in the COPD lung. Grumelli *et al*. [32] also showed that lymphocytes isolated from the lung of emphysematous subjects express higher levels of CCR5, another Th1 receptor, and of CXCR3 than those of normal subjects. Finally, Koch *et al*. [22] found an increased CXCR3 expression by systemic CD8^+ ^T lymphocytes of COPD smokers compared to that of normal smokers. All of this information suggests that systemic T lymphocytes do not show higher levels of activation markers in the blood of emphysematous subjects, but that they may be more sensitive to chemokines induced by IFN-γ, thus facilitating their recruitment into the lung.

One limitation of our study is the relative small number of subjects in each group which may potentially mask a significant difference, and power analyses determined that 37 subjects in each group would be necessary to reach an 80% power. Assuming that our subjects are representative of a larger population and even with significant differences in peripheral T cells perforin and granzyme B expression between normal smokers and emphysematous smokers (means differences < 10%) (see Figure 4), this difference would not be of important clinical relevance and would still give results different from those of Hodge *et al*. [23].

## Conclusion

In summary, this study shows for the first time that, despite evidence of low-grade systemic inflammation, expression of perforin, granzyme B, and FasL by peripheral CD8^+ ^T lymphocytes do not vary between emphysematous smokers, smokers and non-smokers with normal lung function. Our data is in concordance with other studies that have showed that the expression of activation markers by systemic CD8^+ ^T lymphocytes is not different in emphysematous subjects than in normal subjects. Specific recruitment of activated cells by the lung may explain the local cellular inflammatory phenotype of emphysematous subjects. Further studies on T lymphocytes recruitment by the emphysematous lung might be useful to provide a better understanding of the physiopathology of this complex disease.

## Competing interests

MCM and JP declare that they have no competing interest. JM has received unrestricted research grant from ALTANA Pharma and funding for participation in multicentre study from GlaxoSmithKline.

## Authors' contributions

MCM performed real-time PCR analyses, TNF and IL-6 immunoassays, flow cytometry and PCR data analyses and wrote the manuscript. JP participated actively to subject recruitment, performed blood processing and flow cytometry analyses and helped to draft the manuscript. JM conceived of the study, and participated in its design and helped to draft the manuscript. All authors read and approved the final manuscript.
